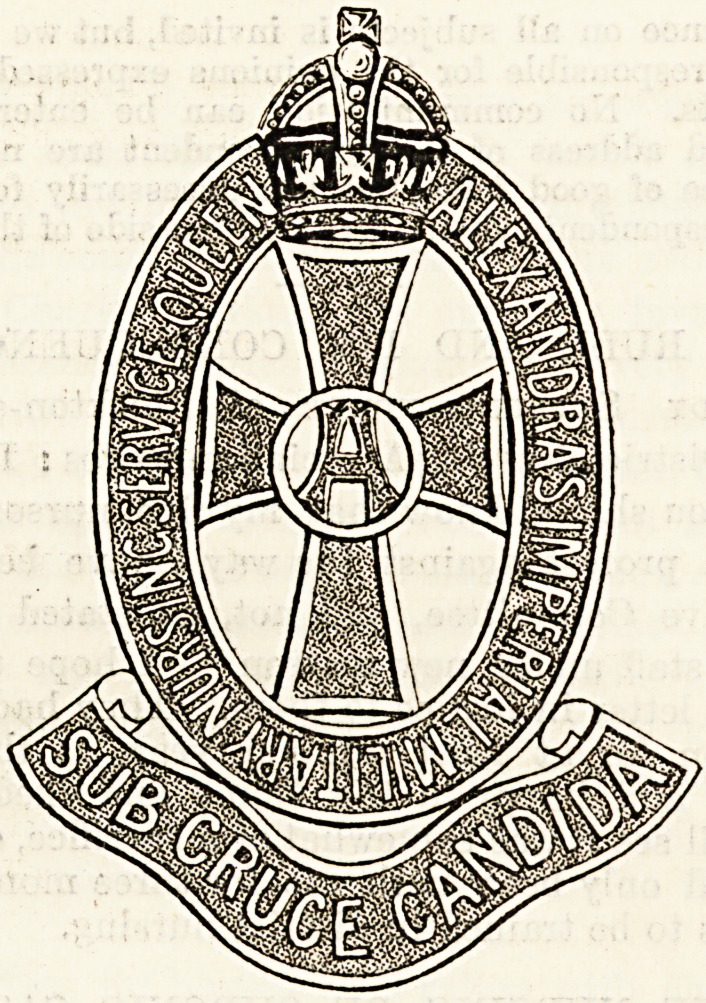# The Hospital. Nursing Section

**Published:** 1905-06-24

**Authors:** 


### The Hospital.

### Nursing Section.

Contributions for this Section of "The Hospital" should be addressed to the Editor, "The Hospital" Nursing Section, 28 & 29 Southampton Street, Strand, London, W.C.

No. 978.—Vol. XXXVIII. SATURDAY, JUNE 24, 1905.

### Notes on News from the Nursing World.

### A MESSAGE FROM FLORENCE NIGHTINGALE.

The opening of the "Nightingale" Nursing Home at Derby on Friday in connection with the Royal Derby and Derbyshire Nursing and Sanitary Association, was marked by an event of great interest. Before the ceremony, which was performed by the Bishop of Southwell, took place, and during the progress of the annual meeting of the members of the Association, Mr. E. S. Johnson, the honorary secretary, announced that a telegram had just been received from Miss Florence Nightingale, which ran as follows, "Florence Nightingale sends cordial good wishes for the future usefulness of the Nursing Home, and prays for a blessing on the work and the workers." Subsequently, at the function in the home, the chairman of the board of management made a brief statement, in which he spoke of the desire to commemorate the life work of Miss Nightingale. The old Derbyshire Infirmary had a "Nightingale" wing, but with the rebuilding of the hospital that testimony to her noble work had been lost. It was therefore felt that advantage should be taken of this opportunity of perpetuating her memory in a suitable and lasting form. He proposed that the best thanks of the assembly be sent to Miss Nightingale for her kind message of sympathy and encouragement in the work of the Association which they had received that day. The motion was enthusiastically adopted. The Bishop of Southwell then formally dedicated the new home to the glory of God and the memory of Florence Nightingale. We may add that before the home was proceeded with the plans were looked over and approved by Miss Nightingale. There are about a dozen beds in the building, which stands in its own grounds, and all the latest medical appliances have been instituted. In the report of the Association it was intimated that the cost of property, alterations, and furnishing will amount to about £5,000. The financial position of the organisation is so prosperous that a special bonus of £365 has been divided amongst the nurses and the staff, and £542 placed to the contingent fund for old and disabled nurses.

### AN AMERICAN NURSE AND THE SELECT COMMITTEE.

The Select Committee of the House of Commons on Nursing will resume their sittings on Tuesday, and we presume that their labours will be finished before the prorogation of Parliament. It is a pity that, meanwhile, Miss L. L. Dock should have considered it advisable to compare the members of the Committee unfavourably with American legislators. Writing to the *American Journal of Nursing*, Miss Dock says that "as a whole they do not compare well with our legislative committees that we have appeared before at home in broad grasp of principles and in quickness of understanding." She then proceeds to except the chairman, who is known to be an advocate of State Registration, and "perhaps three others." She adds, "the rest spend an inconceivable amount of time in higgling over insignificant details and supposititious difficulties, and do not yet seem to have realised that it is an educational question." After this, we are not surprised to hear that Miss Dock's "impression" is that the Committee will not report favourably upon State Registration.

### AN INGENIOUS HOSPITAL MATRON.

It is not often that the matron of a hospital enters upon her term of office at a time when the premises are on the eve of reconstruction. But it must be admitted that Miss A. M. Smith, matron of Tunbridge Wells General Hospital, with whom an interview by our Commissioner is reported elsewhere, made the most of her opportunities. For example, finding that curtains over the nurses' dresses were not sufficient to adequately protect them from dust, and yet feeling that she ought not to ask the committee to go to the expense of new wardrobes, she obtained the services of a handy man, put him in the cellar of the house which was temporarily used as a nurses' home, and having begged for all the odd pieces, she employed him for some weeks to convert the wood into hanging cupboards. When these, as well as the old shabby furniture, were painted white by the same man, they made quite pretty little suites. Another device of the ingenious matron was to utilise a high red screen so as to prevent the beauty of the window of the children's ward being obscured at night. The mortuary will always be a memorial of her practical sympathy for the friends of those who die in hospital. She originated the idea of beautifying an old out-house for the purpose, obtained the necessary money, and succeeded in carrying out her plan.

### AN UNDESERVED CENSURE.

At an inquest on Saturday, held at Westminster, on the body of a four-weeks-old child of Italian parents, it was stated that the mother had been at first attended by a midwife from the British Lying-in Hospital, Endell Street, but afterwards by a "person described as a 'nurse,' who, however had no training beyond that obtained in two months." The evidence showed that the child died from an internal complaint, resulting from improper feeding. The jury returned a verdict accordingly, and expressed the opinion that the parents ought to have exercised more care, and added a rider that the hospital authorities ought to be censured for not having sent a certified midwife to attend the child for at least one week. With regard to the facts of the case, we are informed by the matron of the British Lying-in Hospital that the child was born on May 18th, was not premature, and was apparently strong and healthy. As usual the midwife who had charge of the case visited the mother and child in company with the probationer training to be a monthly nurse for the first three days, the baby being washed and dressed each day for the next two days by the probationer. After that the visits were only every other day till the tenth day, when, if all goes well, it is customary to discontinue them altogether. In this case the baby was apparently quite in good health when the probationer ceased attending, and the mother was feeding it. When, however, the mother and child, in accordance with custom, went to the hospital at the end of three weeks, the matron at once noticed that the baby looked pinched and was losing weight. Questions elicited the fact that the woman no longer fed the baby herself, and was giving it artificial nourishment. The matron told the woman to take the baby immediately to a doctor whom she named. This was not done, and the child died. It is absolutely denied that there was any want of care on the part of the hospital authorities.

### NURSING AND MIDWIFERY IN ITALY.

The question of the present position of nursing in Italy is dealt with elsewhere by a correspondent who has herself been temporarily matron of an Italian orphanage. It will be seen that there is not only room for many improvements, but that those who are engaged in nursing in Italy are not even provided with absolute necessities. The picture drawn by our contributor of her tour of inspection soon after her arrival at the orphanage will, on this point, prove convincing. It is not surprising that, as Italian nurses are mostly women who have had some experience but no training, Italian doctors very much appreciate English-trained nurses. This is one hopeful sign; the other is that Italian midwives are properly trained and generally hold a diploma or certificate from some university. In due time, it may be hoped, the Italian people will realise that it is quite as important to have fully-trained nurses as fully-qualified midwives.

### BELFAST MATERNITY HOSPITAL AND THE CENTRAL MIDWIVES BOARD.

So far as the Central Midwives Board are concerned the facts in connection with the application of the Belfast Maternity Hospital for the approval of its certificate as a qualification under Section 2 of the Act of 1902, we understand, are these. The application was submitted at the meeting of the Board on March 23, 1905, and, after consideration, it was resolved, "that the application be refused, no useful result being capable of attainment, in view of the fact that the privilege, if granted, would expire on March 31." The reply of the hospital authorities is that the new building was not ready for occupation until the end of 1904, that they had informed the Central Midwives Board as to the position of affairs more than a month previously, and that they had received an assurance that the subject would be considered when the new buildings were ready. It is a pity that the application was not made sooner, but it will be a matter for regret if a number of midwives who have been adequately trained and received their certificates from an institution which is now recognised as a training school by the Central Midwives Board, are prevented from practising.

### MORE NURSES FOR BIRMINGHAM INFIRMARY.

Birmingham has not belied its reputation. The recommendation of the Infirmary Committee to spend £3,500 for the purpose of providing adequate accommodation for the nursing staff has been carried, only six of the Guardians voting for the postponement of the scheme. The Chairman of the Committee, in moving its adoption, stated that whereas in a number of similar institutions there is a nurse to every 7·5 patients, the proportion in Birmingham Infirmary is one nurse to every 11 patients. He also explained that the increased cost of the staff is caused by the policy of abolishing the system of taking paying probationers and engaging probationers who receive a salary during their three years' of training. It was asked why it had been decided to do away with paying probationers, and the reply was that "they did not do the work properly." In that case the abandonment of the system in this instance is a wise step, but in many institutions paying probationers are by no means a failure. However, it is satisfactory that Birmingham Infirmary is to be adequately staffed; and we do not think that the gradual increase of the number of nurses from 74 in 1892 to 101 in 1905 need be at all a matter for alarm to the ratepayers.

### DISTRICT NURSING IN PADDINGTON AND MARYLEBONE.

It is much to be regretted that the Paddington and St. Marylebone District Nursing Association, which has now been doing excellent work for nearly a quarter of a century, should be constantly in debt to its bankers. In the annual report, which has been forwarded to us, special attention is called to the deficiency of funds. While there are a great many poor in the area which the Association covers who derive benefit from the ministrations of the nurses, there are also a great many well-to-do persons, and we think that £778 contributed in 1904 by the residents of between 30 and 40 parishes, is by no means a representative sum. It would make an important difference to the receipts if contributions were sent in by the churches generally instead of by three only. But the list of subscribers should be larger, and we suggest that a special effort should be put forward to obtain an increased number of smaller amounts. We believe that if the needs of the Association were brought directly before the wealthy and generous people in the two boroughs there would be no lack of response. The more guineas the better, but why not half-crowns and shillings?

### THE DIETARY QUESTION OF HALIFAX.

The Halifax Guardians, at their last meeting, discussed the proposal of the Hospital Committee, who reported in favour of a revised dietary. Instead of 5½ lb. of meat per week to each nurse they recommended 5 lb., and poultry equal to one chicken for four people in place of three people. They advised the reduction of eggs from seven to five per week, and the provision of fruit to the value of 3d. per week instead of three times a week. An allowance of a pound of fish was also recommended. The difference in the cost of the revised list and the old one was estimated at about £1 15s. per week. This modest proposal met with opposition, one guardian suggesting that the food allowed to the officials ought to be reduced, and "a little bit put on to the dietary of the people in the workhouse." He was not, however, able to carry more than two of his colleagues with him, and the amended dietary was adopted by a large majority.

### GARDEN PARTY AT ST. THOMAS'S HOSPITAL.

Favoured by fine weather the garden party at St. Thomas's Hospital, on Thursday afternoon last week, was an enjoyable function, and the attendance was exceptionally large. Several matrons were among the guests. Nightingale nurses, past and present, compared notes, and old friends made the most of a pleasant opportunity of meeting each other.

### QUEEN ALEXANDRA'S MILITARY NURSING SERVICE.

We are officially informed of the following changes in Queen Alexandra's Imperial Military Nursing Service. Miss H. B. Derby and Miss A. Ayre, staff nurses, have been posted to the Cambridge Hospital, Aldershot. Miss S. K. Bills, Miss B. N. Daker, and Miss G. Knowles, sisters, have been transferred to the Military Hospital, Millbank, from the Cambridge Hospital, Aldershot. Miss A. E. FitzGerald, staff nurse, has been transferred to the Station Hospital, York, from the Cambridge Hospital, Aldershot. Miss M. Kendall, Miss E. M. Pettle, Miss L. A. Rideout, and Miss A. A. Wilson, sisters, are held in readiness for service abroad; and also Miss F. M. MacGregor and Miss L. M. Moor, staff nurses. Miss E. Eardley has resigned her appointment.

### THE NEW MATRON OF NORTHWOOD HOSPITAL.

The appointment of Miss Mary Donaldson as matron of Northwood branch of the Mount Vernon Hospital, Hampstead, is announced. Miss Donaldson, who was trained under Miss Hull at the Great Northern Central Hospital, Holloway, has since been on the staff of the London Temperance Hospital. She first took holiday duty at that institution, and in October, 1903, became home sister. The London Temperance Hospital offers excellent opportunities for any nurse wishing to qualify for a matron's post. There are special advantages when there is no steward, resident medical superintendent, or secretary, for the matron and her assistant to acquire a very varied and minute knowledge of the administrative department of hospital work; and Miss Donaldson will, doubtless, fully justify her promotion to the ranks of the nursing hierachy.

### PAST AND PRESENT NURSES OF THE SAMARITAN HOSPITAL.

After the opening on Wednesday last week of the new out-patient department at the Samaritan Hospital the matron, Miss Butler, who is leaving shortly, gave a farewell tea to her past and present nurses. Tea was served in the consulting room and in recesses of adjoining rooms. The out-patient department had been prettily arranged with flowers and flags, and with its white tiled walls proved a delightful retreat in which to take tea on a hot June day. Regret had been expressed during the opening ceremony at Miss Butler's resignation, and hopes that she might enjoy long life and happiness. With regard to the impending changes nothing definite has yet been settled, but there is one solid fact of considerable importance. Sixteen bedrooms for the nurses have been built over the out-patient department. This arrangement, besides setting free much needed space for patients in the main building, will materially contribute to the comfort of the nursing staff.

### A COUNTY ASSOCIATION FOR BERKSHIRE.

On Saturday a meeting was held under influential auspices at Reading for the purpose of forming a County Nursing Association for Berkshire, to be affiliated to Queen Victoria's Jubilee Institute. Miss Amy Hughes attended on behalf of the Institute, and described, in detail, the work hitherto accomplished as regards the nursing of the sick poor in towns and country parishes, as well as the more extended efforts being made in anticipation of the operation of the Midwives Act. After it had been unanimously decided to form an Association for Berkshire, Mrs. Benyon mentioned that so far no direct appeal for funds had been made, but that she had received spontaneous offers from friends to contribute about £264 in donations, and £115 in annual subscriptions. A number of ladies and gentlemen were then appointed as an executive committee to take all the necessary steps and report to a future meeting of the subscribers.

### A JOURNAL FOR KINGSTON INFIRMARY NURSES.

The first number of the *Kingston Infirmary Nurses' League Journal* has made its appearance, and it is a very creditable production, with a pretty cover, and a full-page picture of the Union Infirmary. The letter-press is clear and well arranged, and there is much matter which will be read with interest by past as well as present Kingston nurses. We observe that the members of the Infirmary League have decided to adopt a bronze badge, with the initials K.I.N.L. on the bar from which it is suspended. If funds permit, as we hope they may, the *League Journal* will be published quarterly.

### SHORT ITEMS.

The late Mr. C. W. Curtis, of Dover, who bequeathed various sums to charities, left £250 to his nurse.—In the statement issued by the treasurer of Guy's Hospital as to the result thus far of the appeal on its behalf, Mr. Bonsor mentions that there have been a considerable number of contributions to the million shilling fund, and says, "this is almost entirely due to the personal efforts of our own doctors, students, and nurses."—Last week at St. Peter's Church, Hereford, Miss King, formerly sister at the Union Infirmary, Leeds, was married to Mr. Gilbert Harding, assistant master at Sunderland Workhouse. Mr. and Mrs. Harding will shortly take up their duties as Master and Matron of the Hereford Workhouse, to which they have lately been appointed.

### The Nursing Outlook.

"From magnanimity, all fear above;
From nobler recompense, above applause,
Which owes to man's short outlook all its charm."

### POST-GRADUATE INSTRUCTION AND PRIVATE NURSING.

The want of some system by which certificated nurses after leaving the training school can have facilities for post-graduate instruction is becoming more and more felt. At the best of the schools experiments have been made by the institution of post-graduate classes and the affording of facilities to certificated nurses, to come back to the hospitals for the purpose of post-graduate instruction, which show that it is feasible, though difficult, to meet the case to a certain limited extent at any rate. At Guy's Hospital every certificated nurse is afforded the opportunity of attending the institution, should she wish to devote herself to private nursing. The present matron has also instituted post-graduate classes. At King's College Hospital, as opportunity offers, facilities are given at the matron's discretion to some of those nurses who wish it, to have the run of the institution for a short time, so that they may see and master any new developments which have taken place since their probationer days. No doubt other nurse-training schools have attempted to help their old probationers to some extent too in the matter of post-graduate instruction, but in reality a definite system will have to be evolved if the demand for post-graduate instruction is to be adequately met. Post-graduate instruction is essential to the well-being of the most competent nurses in their own interests, and in the interests of the public. There can be no question that it would be good for the nurse-training schools and the hospitals, good for the public, and the nurse if a system could be evolved whereby the public can be educated to look more and more to the hospitals for the supply of nurses which they need. The Guy's plan is just to the nurse and advantageous to the public, whilst it helps the institution by keeping the nurse in touch with her alma mater and so promotes *esprit de corps* and good feeling all round.

Post-graduate classes are an excellent and necessary complement to a nurse's education, but they do not entirely meet the position. In addition to the classes it is a great advantage to a nurse to be able to return to her old hospital, and to see for herself exactly what is being done and what developments have taken place since her probationer days. New treatment often involves new methods, and both are matters of supreme importance to the efficiency of the nurse and materially enhance her value to the patient. It is of great importance to the nurses and the institutions where they are trained, that, so long as each certificated nurse continues to pursue her calling, she shall keep so up-to-date in all respects as to occupy the first rank. In this way the reputation of the school and of the nurse are necessarily linked together, and any influences which promote a severance of this co-operation and goodwill, or make for friction are to be avoided and condemned by all intelligent nurses and thinking people, whether the latter are connected with hospitals and training schools or are merely members of the general public. Whatever else may be doubtful, it is at least certain that in this country the wisest and most reasonable solution to most of the difficulties presented in regard to nursing matters can and ought to be met by bringing the nurse-training schools into co-operation, promoting the post-graduate instruction by each school of its own certificated nurses, and securing that the public, when they want a nurse, shall grow accustomed more and more to apply to the nearest hospital, which must make provision to meet the demand to the fullest possible extent.

We are convinced that no one is more likely to take a real interest in the progress and success of a certificated nurse throughout her career than those who have taught and instructed her at the hospital in which she was trained. It is maintained in certain quarters that the authorities of the training-schools in such circumstances could make or mar a nurse's career and compel a nurse to walk in the path laid down by the school. Such suggestions, which are made no doubt for theatrical reasons mainly, owe their origin to the desire of a few people who are not immediately connected with the education and training of nurses in this country, to practically assume the control of all certificated nurses, and to govern nursing education in these islands. To point this out is to dispose of the suggestions finally, for nurses are not likely to rush into the arms of strangers of this type from those of the friends they have made during their probationer days, and whom they have the best reason to honour and to trust. Besides, any plan must of course leave each nurse entirely free, if she chooses to take the responsibility of standing alone when she gets her certificate. If our proposals were generally accepted each hospital would offer every nurse, on granting her certificate, the option of coming upon the private staff. If this offer was not acceptable she would pass out into the world to pursue her profession in any direction which might attract her. She would, however, if she was wise, keep in touch with her old school by joining the League or other organisation in connection with her school, and so be enabled to report her progress and to arrange from time to time to come back to her alma mater for post-graduate purposes. It must be clear, therefore, to every nurse who has the capacity to think for herself, that the scheme we have sketched will not at any rate interfere with the freedom of her action, though it must prove helpful throughout her career.

### The Nursing of Sick Children.

By James Burnet, M.A., M.B., M.R.C.P.Edin., Registrar, Royal Hospital for Sick Children; Clinical Tutor, Extramural Wards, Royal Infirmary; and Physician to the Marshall Street Dispensary, Edinburgh.

### XII.—SKIN CASES.

The subject of diseases of the skin is a wide one, and its full consideration would necessitate more space than we can possibly devote to it. All that will be attempted in this short lecture will be to give a general outline of the subject, bearing specially in mind those practical points in regard to which every nurse must be well informed.

There are certain terms used in speaking of skin diseases with which the nurse ought to be thoroughly familiar. The simplest form of rash is composed of macules, which are merely discoloured spots. An eruption on the skin is said to be papular when the disease consists of elevations about the size of a small pea or less. When these little elevations contain a clear watery fluid they are known as vesicles. The latter, if larger in size than a pea, are often spoken of as bullæ. Pustules are elevations on the skin which contain purulent material. Scales sometimes make their appearance in connection with skin diseases, and are due to a simple shedding of the skin over the diseased areas. These must not be confused with crusts, which are simply the heaped-up products of inflammation. Such are a few of the terms most generally employed, and it must not be forgotten that many skin affections give evidence of more than one of these varieties of eruption.

Sometimes nurses find great difficulty in determining whether a rash is due to some skin disease or rather to one of the infectious fevers. This, of course, is not as a rule a matter of any great moment to the nurse, on whom the responsibility of making a diagnosis never rests; but it occasionally happens that medical advice is not always at hand, and then it may be of considerable importance if the nurse is able to make a tentative diagnosis. Generally speaking, when the temperature is normal the nurse may feel almost confident that the rash is not connected with any infectious fever, but that it is purely a skin affection. If, however, there is vomiting, sore throat, cough, or any other untoward symptom present it may be advisable to isolate the patient until he can be examined by the medical attendant. This is especially to be advised during epidemics of any of the fevers, more particularly in the case of measles, scarlatina, chicken-pox and small-pox. In fact, it often happens that children are brought to hospital for the treatment of some fancied skin disease, and on examination it is found that the patient's temperature is raised and that he is suffering from one of these infectious fevers.

Before referring to the various skin diseases commonly met with in children it may be well if we look for a little at some general points to be observed in their management and treatment. Fortunately most skin diseases are curable, but at the same time many of them are aggravated by or tend to recur in consequence of some peculiar state of the constitution. Again many of them are affected by disturbances of digestion, while in the case of infants the skin becomes exceedingly irritable during the process of dentition. It will often occur that an infant who had been suffering from eczema seemed to be perfectly cured when suddenly the disease became as virulent as ever, and on inquiry as to the cause it will be found that a tooth is being cut. So, too, with nettlerash, or urticaria as it is termed; this affection is often greatly aggravated by some slight gastro-intestinal disturbance and becomes relieved the moment that this is put right. In most cases the diet requires to be very carefully regulated, and milk should form the principal article of food. Constipation must always be avoided, and a daily evacuation of the bowels secured by the use of magnesia, liquorice powder, or a saline aperient.

Powders, ointments, and lotions are generally employed in the treatment of skin diseases. Of these, ointments are perhaps most frequently used. They may be well rubbed into the affected parts or spread on pieces of lint and laid upon the skin. Lotions are often used when large areas are involved, in cases complicated with itching, and in the treatment of affections of the scalp. Powders are most commonly used where there is irritation of two skin surfaces which are in apposition, such as the region of the groin, axilla, and also the region behind the ear and the folds of the neck. Many skin diseases are of an infectious nature and require the use of antiseptics in their treatment. The latter may be incorporated with lotions or ointments, as found most convenient in each particular case.

One of the most frequently occurring forms of skin disease met with during the periods of infancy and childhood is eczema. This may assume quite a variety of forms, and may appear on any part of the body. Not infrequently it attacks the face and scalp of nurslings. It begins in the form of a few scattered spots of redness which presently run together until an area of considerable size is involved. Blebs form, which eventually burst, so that the surface becomes moist. This discharge then dries up, leading to the formation of crusts. The essentials in the treatment of this disease may be very briefly stated. In the first place all crusts must be got rid of. This is a rule which holds true for all affections of the skin in which there are crusts or scabs present. These crusts may be removed most readily by the application of starch poultices. These may be made as follows:—A teaspoonful each of cold water starch and powdered boracic acid are mixed with a little cold water into a paste. A pint of boiling water is then added and the mixture stirred thoroughly. When cold the thin starch paste so prepared is spread upon cotton and covered over with muslin.

When the crusts have been removed a suitable ointment or lotion may be applied two or three times a day. In the case of the face a mask of lint on which the ointment is spread may be placed over the patient's face, apertures being made for the eyes, nose, and mouth. The child must be kept from irritating the affected parts by scratching, and it may be necessary to fasten the arms to the sides or to apply pasteboard splints to them. The eczematous areas must on no account be washed with water as this prevents healing of the affected parts. It is, however, allowable to use oatmeal water or starch water for purposes of ablution.

Psoriasis is a curious condition characterised by clearly defined areas covered over by fine silvery scales. It may be treated by means of preparations of tar, and also by immersion in alkaline baths. Crysarobin is often ordered by the physician, and the nurse should bear in mind that this substance is very irritating to the skin and should always be applied by means of a piece of flannel.

Ringworm may occur either on the head or on the body. It is due to the action of a parasite. If on the scalp this disease is apt to prove most intractable. The hair should be cut wide of the affected patch which must be scrubbed thoroughly every morning with soap and water, and thereafter a suitable ointment or lotion applied. The child must have a towel and hair brush kept for his own especial use, as ringworm is a disease which is very readily conveyed from one child to another.

Lousey heads are well known in hospital practice and are best treated by the application of a paraffin soak to the scalp and, if necessary, cutting the hair short. An antiseptic ointment, containing sulphur and mercury, will often be found helpful. Such children usually require tonic treatment as well.

The itch, or scabies, is a common skin condition in children, and one which gives rise to intense itching, especially at night. The application of a weak sulphur ointment is generally all that is necessary locally, and this together with thorough bathing of the skin and attention to the child's clothing will usually bring about a speedy cure.

Nettlerash, otherwise termed urticaria, is a disease characterised by the presence of a widespread papular eruption associated with considerable itching. It is generally due to errors in diet and to constipation. Alkalies internally, together with a calomel purge, and the external application of a soothing lotion generally suffice for its relief. It is, however, very apt to recur from time to time, and in some cases proves somewhat intractable.

Such are some of the commoner skin diseases, and those which nurses most frequently have to deal with. The rarer affections, not being of everyday occurrence, do not require any consideration at present. For information regarding these, the nurse must refer to special treatises on the subject.

### The Nurses' Clinic.

### THE DISPENSARY. BY A CERTIFICATED DISPENSER.

### POWDERS AND CACHETS.

Substances which are ordered to be taken in powder form are generally kept in stock ready pulverised; sometimes, however, the physician may order crystals, and occasionally, though very rarely, roots, leaves, or herbs. In the latter case the grinding should be performed with an iron pestle and mortar, as considerable force will be required, which might crack thin porcelain or wedgwood ware. When sufficiently ground the particles should be passed through muslin or a sieve to ensure there being no coarse pieces left. Crystals must be very finely ground, and when two substances containing large proportions of water of crystallisation are required, as alum and acetate of lead, they must be ground separately, and then lightly mixed together with a spatula. Quinine salts must be carefully rubbed to a powder.

Hygroscopic and volatile substances should first be wrapped in white paper, then in a second covering of waxed paper. Carbonate of ammonia should be wrapped in this manner and sent out in a bottle, as it is very deliquescent.

Occasionally a powder is ordered in bulk, the patient being told to apportion the dose; in this case it should be sent in a wide-mouthed, well-corked or stoppered bottle with the directions plainly written on the outside. Gregory's and liquorice powder or ginger are often prescribed in this way. If the powder is for external use or to be made by the patient into a lotion, a "caution" label must be affixed, and it must be sent out in coloured paper. When six or eight or more powders are ordered they should be all folded exactly the same size and sent out in a cardboard box; any number under half-a-dozen can be sent in an envelope. Powders to be taken in a state of effervescence must be separately wrapped in white or coloured papers, the alkaline substance in blue and the acid in white. Seidlitz-powders are ordered in this manner; they are composed of Rochelle salt and bicarbonate of soda with tartaric acid. When prescribed to be sent ready mixed the acid and alkaline ingredients should be separately dried at a low temperature, then mixed lightly in a mortar, and sent out in a bottle. If the temperature at which they are dried be too high the moisture is liable to be reabsorbed; it should not be higher than 90° F.

Great care must be taken in weighing powders; sometimes the physician orders the amount for one powder only, directing what number is to be sent; thus in this case a powder containing four grains of one substance and six of another, and 12 of such to be sent, the dispenser would require to weigh twelve times more than is ordered. There are different ways of doing this; it can be done either by weighing the amounts for each powder separately, which is the most accurate, but a very tedious way, as in the case of a dozen powders with two ingredients in each it means weighing out 24 separate times. Another manner of weighing, which should only be attempted by an experienced and expert dispenser, is to weigh at once the whole amount required for the 12 powders, first of one ingredient, then of the other, put them both on a piece of paper and thoroughly mix them; next divide the whole exactly into two heaps so that there will be the amount for six powders in each heap; divide these again into two, so that each will contain the amount of three powders, which are then divided equally: a little practice will enable the eye to judge very correctly whether they are all the same weight, and one or two can be tested on the scales to prove whether they have been accurately judged. This plan, however, must never be adopted when a poison is one of the ingredients. In dispensaries when there are a great many patients and many children who are ordered a small powder, perhaps a grain or two of Hyd. c̄ Cret. every night, the seven powders are sent in two packets with the directions that they are to be divided into three and four doses respectively; this, however, is not a good plan, and not one to be recommended, although it saves a certain amount of labour; it is best and safest to make in leisure time the powders most frequently required to be kept in stock until ordered. It often entails, too, a

### THE NURSES' CLINIC—*Continued.*

tiresome calculation, and so in the end does not save a great deal of time, as when, for instance, 25 grains are prescribed to be sent out in two packets—one of four and the other containing three doses. The greatest care must be taken in reading a prescription to notice whether it is written for one powder only or to be divided into a certain number, or a terrible catastrophe might ensue if the amount written for half a dozen powders were put into each one, the dispenser multiplying by six instead of dividing.

Powders should always be very carefully mixed together, especially when some of the ingredients are coloured and some are white, as the patient is not favourably impressed if he notices different shades of colour here and there, proving that it has been carelessly or hastily done. When only one powder composed of two or three ingredients is ordered, it is best to mix it on the paper itself with a spatula; but for larger quantities the mixing should be done in a mortar, the most inactive ingredient taken first and the more active added to it and the whole thoroughly mixed. If a very little of a very active substance be ordered, it should be put on to a few grains of the less active and incorporated before adding the rest gradually. The matter of folding powder papers is generally found very difficult by beginners, but a little practice will soon overcome it. The simplest plan for small powders is to have the papers cut to about four by five inches; they are folded lengthways to within half an inch of the top, which is turned down, thus forming a flap; the flap is again turned down to half-way across the packet; the cardboard box they are to be sent out in is measured for the length, and the ends of the paper are turned underneath evenly to the right length to fit comfortably into it. It is best to smooth the papers with a spatula or paper knife to prevent creases, and again after the powders are in, to prevent their bulging in the middle, leaving the ends flat. It has been found a good plan to keep papers ready folded to be used when wanted, as the powder is less likely to be spilled on being put into ready creased paper than when the paper is being folded, especially by novices. A more recent and far nicer way of administering powders is in the form of cachets, when the drugs are enclosed in a thin wafer paper cell, and swallowed with water like a pill. They can be made in all sizes to hold from one to 24 grains. There are six sizes of varying depth and diameter. The cachet machine is made up of several pieces, the body is composed of three plates on hinges; a funnel, "thimble," and a roller made of felt, for moistening the rims of the cachets, are also required. Great care must be taken over the moistening, if they are damped too much the rims may be damaged, and if too little they gape open. Cachets are sent out in boxes.

### The Nurses of Tunbridge Wells General Hospital.

INTERVIEW WITH THE MATRON. BY OUR COMMISSIONER.

Travellers along the road from Tunbridge Wells to Southborough have, year by year, grown accustomed to see the rugged face of the old General Hospital standing close upon the street, solid, useful, but certainly not ornamental. The face remains unchanged, but the interior has of late as completely altered as if at the wave of a fairy's wand a transformation scene had been effected.

"But," said the matron, Miss A. M. Smith, when I remarked upon the metamorphosis, "the transition has been a long and a tedious one, and occasionally some of us fancied for just a few moments that we should never get straight. You see the old hospital itself has been turned into the nurses' home, whilst the out-patient department, and all the wards are new. During the building the nurses had to be lodged in houses near, and there were other tiresome makeshifts; but that is now a thing of the past, and when you have seen it I think you will agree that—thanks to the committee, who have been most good in trying to give the best possible both to patients and nurses, and to our architect, who has been both clever and resourceful—our hospital will bear comparison with any."

### The Increased Accommodation.

"You and the alterations came together, did they not?"

"Building had just started when I was made matron, now nearly two years back. All my experience before had been at Charing Cross Hospital, where I was trained, and worked gradually up till I became the temporary matron for six months between the illness of Miss Gordon and the appointment of the present matron, Miss Heather Bigg."

"Are the new wards here larger than the old?"

"Yes, and they are an improvement in every way; better built, better ventilated, and capable of holding 20 more patients. We can now take 70 patients; a large men's ward of 24 beds, and one little ward with a single bed; a large women's ward of 24 beds, and one single bedded ward; a children's ward holding 16 cots, and an isolation block with two beds and two cots."

"When you increased the number of your patients I suppose you had also to increase your staff?"

"Yes, three more probationers were added. I now have 15 probationers and four sisters."

"Did you find many changes necessary when you came?"

"No; I have been exceedingly fortunate. One of my sisters has recently accepted a position as matron to the Tunbridge Wells Eye and Ear Hospital, but this is the first of my staff who has left since my arrival. Of course I was able where needed to reorganise quite naturally, as it were, for all the outward conditions having been remodelled, the rules and regulations of necessity followed suit."

### The Probationers.

"Will you tell me some details about the probationers?"

"To begin their training they must be between 21 and 30 years of age. I never under any circumstances accept a girl less than 21, which I consider quite young enough, and I am very firm on this score. The social standard of the girls who apply has a tendency to go upwards, and I am glad that it should be so. They receive no salary the first year, £8 the second, and £12 the third. They are also given the material for their dresses, caps, and aprons for indoor uniform. They wear, the first two years, striped galatea dresses, and the third plain grey. No outdoor uniform is required. There are no conditions as to religion, but I make a point of ascertaining to what community the nurses belong, and do my best to see that they go to some place of worship on the Sundays."

"Do you promote your nurses later to be sisters?"

"No, the sister of the children's ward and the theatre has been trained here, but the others have all been appointed from outside, and I much prefer it. Their salary is £30, rising annually to £36."

"Do you have many serious cases?"

"Yes. During the last quarter there were 140 operations, of which 27 were abdominal sections, and there are a good many gynæcological cases. The out-patients' department, too, is unusually large, and the new buildings we find a great boon. Twenty-seven thousand visits were paid by out-patients last year. As to the work of the sisters, the theatre sister is also sister of the children's ward, two other sisters have charge, one of the male ward, one of the female ward, and the fourth is night superintendent."

"How soon do the probationers begin night duty?"

"At present they have to go on night duty during their first year, but this I hope to change before long, and to arrange so that they shall have no night work till they enter their second year. Now they have three months in each year, nine months in all during their three years' training. There is one probationer in each ward, and the night superintendent is over all. The probationers do not go into the theatre during the first year, but work for four months in the men's ward, four months in the women's ward, and four months with the children. During the second year they have further experience in the out-patients' department, whilst a third-year nurse takes charge of the out-patients' department and is also theatre nurse. This is generally during her last three months. When the nurses leave here they usually go as staff nurses in a provincial hospital, or take up private nursing."

"Of course the nurses have lectures?"

"The first year I give them lectures on practical nursing, on which they are afterwards examined. The house surgeon lectures to them the second with another examination at the end, and there is a final examination of course before they are given their certificate at the end of the third year."

"What time do they go on duty?"

"They are called at six, have breakfast at 6.30, and go on duty at 7. They have two hours off duty, either morning or afternoon as is most convenient, and four hours on Sunday for service, a day a month, and three weeks' annual holiday. The nurses have supper at eight, followed by prayers which are held in the nurses' sitting-room. Then they go back into the wards till nine o'clock. The sisters do not come on duty till 8 A.M., and they leave the wards about the same time as the probationers.

### The New Hospital.

"And now," said the Matron, "I expect you will like to see over the hospital."

So leaving the sitting-room appropriated to the Matron, quite charming with its artistic green walls, its yellow-tiled fire-place, and its comfortable chairs and cosy cushions, we crossed over from the nurses' home to the hospital itself. This is reached by an open corridor with tessellated floor, which the nurses prefer in fine weather. When needed, a covered way beneath effectually protects them from wet or cold.

The wards are delightful, white washing walls with art-green tiled dados, and red blankets to give the necessary brightness. The tone of harmony in the whole is accentuated by the window blinds, which are made exactly to match the dado in colour, the tiled stoves being of the same hue. The pureness of the atmosphere—which is yet not cold—is secured by air continually passing in from outside over the hot-water pipes. There is ventilation under every bed, the warm fresh air constantly circulating. The beds are new Lawson Taits, and the Matron said "We have succeeded now in giving every patient in the hospital a feather pillow—a great desideratum, I think."

### Features of the Wards.

The Matron also especially drew my attention to the three-cornered cupboards in the lavatories, where necessary utensils are kept. To ensure perfect disinfection, two sides are open to the air by means of gratings, the glass door forming the third. The upward flush in the sink for bed-pans lessens the nurses' work, whilst making it more thorough. As we left the ward I remarked on the balcony where any patient developing a tendency to phthisis can live and sleep till he is removed to a sanatorium, and the day-room, which leads off the ward, for such patients as are well enough to be out of bed. Here stand a harmonium and two litany stools which are wheeled into the wards when the clergy hold their services there.

Then we passed on to the single ward, occupied at that particular time by a nurse who had a touch of influenza, and after a peep at the sister's ward-sitting-room (each ward has a small one), the matron showed me the leads which are railed in where the men, when convalescent, are allowed to smoke. Down the teak stairs we came to the operating theatre. Here the radiators for heating purposes are all on hinges so that they can be swung out to be nearer the patients and to allow of thoroughly cleaning behind them; the taps for hot and cold water are turned on or off by a movement of the nurse's elbow and the Berkfeld filter is also in use here.

### The Children's Ward.

The children's ward, built in commemoration of Queen Victoria's Jubilee, is one of the prettiest in England. The walls are made entirely of painted tiles illustrative of nursery rhymes, each picture being three by nine feet. These have

Children's Ward, General Hospital, Tunbridge Wells.

all been presented to the hospital, "Ride a Cock Horse" having been subscribed for by the very poor of Tunbridge Wells. The cots have also all been given by the children of the place; the sides are movable and when lifted slip automatically underneath the mattress so that they are at once out of the nurses' way. The window at the end of the ward is in shape like a half-open four-fold screen, and it is of such a pretty shape that the Matron felt it a shame to cover it up even at night. So instead of blinds to match the dado as in the rest of the room this window has only a tall red screen to stand in front of it at night, high enough to shut out inquisitive eyes, but low enough to show the pretty tracery of the window above. Against the wall I noted three wicker chairs upholstered in red—small, smaller, smallest—exceedingly suggestive of the story of "The Three Bears," and making delightful resting places for convalescent tots, who have also a big leads to play on when they wish.

### The Mortuary.

Before we went back to the Home, the Matron took me out to the mortuary. As we crossed the courtyard she explained that the old out-building which was originally used as a dead-house had been so bare and hideous that it distressed her much to have to take the bereaved friends into such a place. She mentioned this to one of the committee and he promised her £10 to improve it, but unfortunately, he died before he could give the money. Then she feared

### THE NURSES OF TUNBRIDGE WELLS GENERAL HOSPITAL—*Continued.*

her dream was over, but some of the gentleman's friends, hearing of the incident, made up the amount, and the result is a beautiful little room. The tiny table with its cross and its brass vases full of white flowers (to provide which there is a small permanent subscription fund amongst the nurses), the walls painted in soft colours, with the text "I am the Resurrection and the Life" emblazoned on the side, and the sheet with the red cross worked upon it, all speak silently of loving sympathy.

"And," continued the Matron, "when we see how the relatives are touched when we bring them here, we feel amply repaid for any little additional trouble."

### The Nurses' Home.

Eventually, we came back to the Home which was only completed last January. In the nurses' sitting-room a nurse was singing whilst another played her accompaniment. The pleasant dining-room was unoccupied, but the Matron told me with a smile that it used to be the old operating theatre, and that some visitors wondered how nurses could eat their food in a place with so many gruesome associations! Next we visited the bedrooms. Most of these are separate, but some—those occupied by the junior nurses—are called "cubicles," but the only justification for the term is that two bedrooms have the same entrance door. They are quite spacious, and the inner room opens out of the other, the partition not reaching to the ceiling as do the outside walls. The furniture is all white, and ample for the purpose. The nurses are indebted for their hanging cupboards to the Matron's ingenuity. In their old quarters their dresses, etc., were protected only by a curtain. But though the Matron felt she could not ask for new wardrobes, she was anxious these curtains should be done away with; so whilst in the temporary home, she secured all the pieces o wood which were not wanted, had them made into rough cupboards, and for some time employed a man in the cellar of the house to enamel them with white paint as well as all the old, rather shabby, furniture, one piece at a time as it could be spared. There are six bathrooms in the Home, two for nurses, one for sisters, one for matron, one for servants and one for the house-surgeon.

### The Sisters' Quarters.

The sisters' quarters are entirely distinct from those appropriated to the nurses, and the sisters have their tea and supper in their own sitting-room. On this subject the Matron mentioned that she was desirous as much as possible to separate the sisters from the nurses. She found it so much better for discipline, and she considered herself very fortunate that one corner of the Home contained just the needful number of rooms, even including a bedroom for the night superintendent at the extreme end of the passage. The top floor of the Home is devoted to the maids and the night nurses. Before leaving, the Matron showed me the isolation block, which occupies the opposite end of the building to the nurses' quarters, and is quite complete in itself, with a passage between, in which there is always a cross-current of air. All occupants are strictly cut off from the hospital world. There are two little cots and two beds, a small kitchen with a gas-stove overlooking the ward where the nurses cook, and a nurse's bedroom. The isolation when an infectious disease developed not long since in the children's ward was so successful that no other cases followed, and I can believe that the nurses quite enjoyed the temporary occupation of the self-contained flat, where they might almost have imagined themselves enjoying future promotion in a wee cottage hospital of their own!

### Nursing in Italy To-day.

BY AN ENGLISH MATRON.

Nursing in Italy is very different from nursing in England, and an English nurse has to learn to do the best she can without appliances, both domestic and surgical, which she has hitherto considered as essential to the well-being of her patients. At least such has been my experience while acting as temporary matron of an Italian orphanage. I often look back to the old hospital days and wonder what an English doctor would say if he could see me dressing wounds under certainly unusual conditions.

### The Absence of Necessities.

Such necessities as lotion-bowls, mackintoshes, and even bandages are conspicuous by their absence. A tour of inspection soon after my arrival revealed the fact that the antiseptic lotions were contained in flask-bottles similar to those in which the wine of the country is sold, and the aforesaid bottles reposed tranquilly in a clothes-cupboard without even the precaution of lock and key. Such items as minor dressings and ointments found a resting place upon a top shelf of the same cupboard. This arrangement struck me as rather odd at first sight, but further shocks awaited me in the near future. I soon found that it was quite impossible to carry out my cherished hygienic theories in a country where, to put the matter mildly, the sanitary arrangements leave much to be desired, where also unholy smells seem to be regarded with perfect indifference. The bath-room, such as it is, serves in a dual capacity, and is a dark hole at the best. An old zinc bath has to do duty for the whole establishment, and all the hot water has to be brought up a double flight of marble steps by the kitchen-maid. This unfortunate young person recently scalded her face and neck, more or less severely, by dropping the steaming cauldron on the steps. We have neither gas nor electric light in the building, and it must be confessed that lamps and candles have serious drawbacks where children are concerned. Ventilation is but little understood in Italy. The windows are made to open in the middle in the French style, so that if they are left slightly open there is always a danger of their being blown violently apart by the strong winds that often arise suddenly in the night.

### The Small Patient.

During the past winter the children have suffered severely from chilblains. I have tried to account for this fact, but can only come to the conclusion that the want of fires and systematic exercise, coupled with inherited weakness of constitution, is the cause of it. My sitting-room, being the only apartment in the institution with a fireplace, excepting, of course, the kitchen, has had to be used as a surgery, and thither every day during the cold weather streamed a regiment of small patients requiring surgical treatment. We have cases of rickets, persistent anæmia, and a certain number with a tendency to tubercular disease. The latter is a very common complaint in Italy among the poor, and when one considers the overcrowding—a whole family frequently sleep in one room—the want of ventilation, and the dirty habits of the peasant class, it is not to be wondered at. I have been told by a doctor that it is practically impossible to convince the Italian poor of the necessity of disinfection of their rooms in a case of illness. A consumptive patient expectorates upon the floor, and, in fact, anywhere and everywhere, with frequently the most disastrous results to the members of the family, etc., but as ignorance and prejudice generally go hand in hand, hygienic ideas are only instilled with difficulty among the poor.

An Interesting Case.

So far my most interesting case has been a child with severe symptoms of scrofula. She has had a persistent discharge from the nose, which has required a daily flushing with an antiseptic solution for at least three months, accompanied by a discharge from the ear, and ophthalmia. This was followed by eczema of the scalp, which received long and persevering treatment. At the same time obstinate sores on one hand and foot were being daily bathed and dressed with mercurial ointment. Just when we were wondering how much longer the patient would require treatment, she suddenly developed erysipelas of the face, with a temperature rising to 104°. An antiseptic lotion applied to the scalp prevented the spread of the infection, and the use of ice both internally and externally in a few days reduced the temperature considerably. The doctor's prophecy that the erysipelas acting as a "sfogo" or safety valve would probably end all the other troubles has been fulfilled, and the patient is now apparently in better health than ever she was.

The Question of Diet.

With regard to sick diet, Italian children are so unused to drink milk that when they are ill they positively dislike it, and it becomes a question of disguising the flavour in some way or other. They will take broth in any form, and so one has to compromise matters by strengthening their soup. A great deal of my work consists in preventive treatment, prompt attention to sore throats, indigestion caused by insufficiently cooked food, treatment of thread-worms which arise from the same source, and attention to poisoned fingers, which, if not promptly treated, so often lead to very serious mischief. The Italians as a nation suffer from gastric troubles, and to me it seems a natural result of their habit of eating food such as rice, macaroni, vegetables, etc., cooked less than we consider necessary in England. They are also very fond of condiments in the way of strong flavours, which certainly tend to weaken the digestive powers of the stomach. Their faith in the sustaining power of soup, or "minestra," as they call it, is very difficult to shake, and this is only natural, as they never miss a day without partaking of one "minestra" at least. I recently visited an Italian pastor who was suffering from "mumps." His wife was trying heroically to obey the doctor's instructions with regard to liquid food, but only with the greatest difficulty could she induce her husband to touch the milk and eggs she offered him. He told me with the air of a martyr that he had eaten nothing for three days, meaning that he had missed the customary rice and macaroni salad, etc. My patients take very kindly to cod-liver oil, from which they derive great benefit. It is customary in this country to administer the medicines mostly in the form of powders wrapped in a "bolus;" they are much less nauseous taken in this way, especially where children are concerned. In the case of adults medicine is frequently administered hypodermically, or by "punture," as they call a puncture here, probably with the idea of insuring a more rapid effect.

Nurses and Midwives.

I may mention that Italian doctors very much appreciate the services of an English-trained nurse, their own nurses are mostly merely women who have had some experience but no training. The midwives, on the contrary, are properly trained and generally hold a diploma from some university. They are sometimes available for a case of general nursing. District nurses are an unknown quantity in Italy except in the case of two or three Protestant Deaconess Institutions. In the large town from which I am writing there is a civil hospital nursed by nuns, whose management evidently leaves room for improvement, judging from the state of the wards at the time of my visit, but the military hospital is, I believe, a model of efficiency. There is no children's hospital nor any home for paying patients. In Florence and Naples a few nurses are being trained, though not quite on English lines; but Italy is still a young country and has to reckon with a good deal of prejudice, especially where the independent action of women is concerned.

### The Incorporated Society for the Higher Education of Nurses.

The Board of Trade, at the request of the promoters, have delayed giving their decision on the application of the above Society for a license, pending the report of the Parliamentary Committee on the Registration of Nurses now sitting.

### Presentations.

Cumberland Infirmary, Carlisle.—Miss Clare Richardson, on leaving Cumberland Infirmary to be married, after being ward sister and night sister, was the receipient of the following presents, as well as a number of personal gifts from the nursing staff: Antique brass gong and Wedgwood plate from the matron, a silver "James" tea-caddy and spoon from the nursing staff, a set of silver buttons from the lady dispenser, an edition of Tennyson from the resident medical officers, and a gold and torquoise neck-chain from the domestic staff and porters.

### A New Book for Nurses.

Hints to Nurses on Tropical Fevers. By S. F. Pollard, Sister, Army Nursing Reserve, late Sister at Guy's Hospital. Scientific Press, Limited. Fifty-seven pages.

The first chapter of this useful little book is devoted to the equipment of the nurse going out to the tropics, and the care of her own health. Light woollen undergarments are *de rigueur*; gloves and boots should be kept in tin-lined boxes. Warm clothing for the journey there and back and for the cold season, an ætna for boiling suspicious milk and water, a plentiful supply of soaps, and other needful articles which can only be obtained at exorbitant prices out there, must not be forgotten. In the chapter on malaria, stress is laid on prophylactic measures like the necessity of the mosquito net, and of having no standing water, flower or vegetable beds, near the windows. Dysentery is next discussed, and here the chief items of treatment are rest, warmth, drugs—such as ipecacuanha, calomel, and opium, and others, and a diet, consisting of rice or albumen waters, thin chicken soup, etc. The after-effects of dysentery are often serious, most dreaded of all being the liver abscess which may either be detected in time and operated upon, or burst into the stomach, lungs or other parts. In subsequent chapters, cholera, bubonic plague, beri-beri, yellow fever, Malta fever, dengue, prickly heat, typho-malaria receive attention, and we find some valuable hints on diet, prophylaxis and the characteristics of each of these diseases. The nursing literature is so far very poor in books on tropical diseases, therefore we welcome Miss Pollard's volume as a valuable addition to our library. The distinguishing features of each individual tropical disease are described in a clear, concise manner, and will no doubt prove very helpful to any nurse undertaking work in the hot climates, and interesting to those at home. The actual nursing of the diseases, however, has had to take rather a secondary place.

### A Book and its Story.

### A RUSTIC IDYLL.*

Mr. Metcalfe in his careful analysis of the character of Hugh Larkom, the village blacksmith, and of other well-drawn village folk in his Dorsetshire tale, "Peaceable Fruit," proves himself to be a writer of ability with real artistic perception. The characters, few in number, are placed in natural and homely environment, and each is a lifelike study. The dialogues are for the most part written in Dorset dialect. But this, instead of being, as is so often the case, a drawback, is used by the author with intelligence and familiarity, and so adds to the local value of this very well written and charming story.

Wildacres is the name chosen by the author for the village in which the drama of "Peaceable Fruit" was played. In the prologue he describes Wildacres and the district in picturesque language but with such minuteness that to dwellers in Dorset the exact spot would not be difficult to identify.

"Wildacres, indeed, lurks as coyly in the shelter of its trees as any shy Hamadryad of old time. One might cross Table Head daily, winter and summer, and never have any idea of its proximity. For on the south it is effectually screened by the steep escarpment of the hill—a towering wall of sandstone so covered now by birch and hazel as to bear the semblance of a shimmering green cascade. East and west it is shut in by Lord Axminster's preserves; only upon the north side is it free to peep out modestly into the blue distance of the vale. It is doubtful whether in all England there is a more sequestered spot than this. Its population musters only 70 souls, bestowed among 11 cottages; yet quiet as is their habitation, remote from all stress of competition, the people who call Wildacres their home know as much of joy and sorrow, of hope and regret, as any of us more or less favoured folk outside. . . . Of mental analysis and of psychology they are entirely ignorant, and are probably so much happier thereby. But they have their comedies and tragedies set, it may be, upon a tiny stage, but still complete from prologue to epilogue, and embracing every shade of human emotion."

Hugh Larkom, the hero of "Peaceable Fruit," is a strong study, illustrating throughout the truth that true blessedness lies in giving, not in receiving. He was left with a younger brother, some years before the story opens, in possession of the little homestead which they had known as home from their childhood. Here he and brother Charles lived. Charles was easy-going and careless, and lacking in all the finer instincts which dominated the character of the elder brother who is graphically described in the following passage: "A broad-shouldered man, with close-knit frame, small-loined, and deep-chested; brown hair, curled crisply round a sunburnt face; honesty shone from out his hazel eyes; he might have stood as a model of the clean living, healthy yeoman class that won glory for England at Agincourt, and has done nought to tarnish that reputation since. Habitually Hugh Larkom's face wore a somewhat sad expression, born not of discontent, but of unsatisfied ambition. He had the seeing eye and a soul responsive to external beauty; but he had also a humble consciousness of his own limitations. He possessed the imagination of the poet with all its influence for purity and noble deeds, but owing to the conditions of life among which he was placed he was inarticulate, dumb, while he yearned to voice the vague aspirations that filled his being. His was not the happiest temperament, but it is well for the world that it is not so rare as some suppose.' Hugh had for some time cherished a secret affection for a girl known to the brothers from her childhood. She was the belle of the countryside and not without avowed suitors, among whom Charles Larkom was specially favoured. "In outward appearance Charles Larkom was very much like Hugh, but with a weaker and more sensuous cast of countenance; in spite of its weakness, however, the face was of a type that appeals to many women, and in the clear atmosphere of the summer night it showed like a cameo finely cut. He had some local reputation as a humorist—a dangerous reputation for any man not essentially virile and robust." The two become engaged, and Hugh at once gives up all thoughts of a future in which Nellie Miller should move by his side as a wife; but he could not so easily relinquish the day-dreams which he had woven of her as the angel in the house.

"Nellie waiting for him—a wife ruling with the sceptre of love over the small kingdom where his mother had ruled with such sweet dignity for so long! Hugh could picture it all so clearly; Nellie flitting with proud sense of proprietorship about the garden as light and gracious as the butterflies that wavered over the flowers. . . . For days he went heavily seeking comfort and finding none, until at last it came to him by inspiration. 'Some give and some receive,' he thought, and of the two the former are the most blessed; and some give up. The idea stuck in his mind, repeating itself with musical rhythm on his anvil in the forge. Nature was stern and relentless, heedless of the individual, heedful only of the type; but as one progressed, self-preservation ever became less paramount a duty, until in the Perfect Life the lesson of self-sacrifice was taught instead." Hugh carried out to the uttermost the inspiration given for the sake of the woman he loved, never realising the irony of the situation. "Humanly speaking, his love was perfect; but inasmuch as she on whom it was bestowed so lavishly had not loved the highest when she saw it, she doubtless was not worthy of the blessing. . . . So loyal was he to the idealised Nell that it never occurred to him that she might be commonplace as well as Charles." Charles had many ways of employing his leisure hours—hours all too many in the calendar of his working week. His evenings were usually spent at the village inn, and his nights, too often, lurking in Lord Axminster's preserves. Even the near approach of his wedding did not interfere with this last-named occupation, utilised as a means of replenishing his exchequer, unknown to Hugh. On the eve of the wedding day, his absence causing some anxiety to his fiancée, Hugh undertook to search for him, and in consequence came upon him in a poaching affray. Being discovered, Charles had, in self-defence, struck and apparently killed the head keeper. Hugh allows Charles to run off, and takes his place as the culprit. He is taken up and awaits his trial at the quarter sessions. In the meantime the wedding takes place. "What the trial would disclose Nellie could not suggest. At all events she was not sufficiently original to contemplate the postponement of her wedding. . . . Prettier maiden never entered the village church to leave it a happy and hopeful bride; handsomer bridegroom never stepped more proudly down the nave with his wife upon his arm." The history of Hugh Larkom's devotion and self-sacrifice to the worthless brother and his wife must be read in the book itself. But among the many sketches of rural life that have been produced recently nothing better has been done than this one of "Peaceable Fruit," by Mr. Cranston Metcalfe.

* "Peaceable Fruit." By Cranston Metcalfe. Melrose. 6s.

### Everybody's Opinion.

[Correspondence on all subjects is invited, but we cannot in any way be responsible for the opinions expressed by our correspondents. No communication can be entertained if the name and address of the correspondent are not given as a guarantee of good faith, but not necessarily for publication. All correspondents should write on one side of the paper only.]

### A RULE AND ITS CONSEQUENCE.

THE LADY SUPERINTENDENT of Stockton-on Tees and Thornaby District Nursing Association writes: I think it only right that you should know that my five nurses resigned on June 9th in protest against the way I have been treated by the Executive Committee, and not, as stated elsewhere as "change of staff under new matron." I hope that you will publish this letter in justice to them. They had all decided to remain on for at least another year, as they were very comfortable and happy in the Home and liked the work, so that you will see it was somewhat of a sacrifice, especially for one who had only been with me for three months and who was anxious to be trained in district nursing.

### THE NURSING OF CHRONIC CASES.

"E. A. L." writes: I quite agree with the opinion and feeling of "H. S." on the nursing of chronic cases and hope that the nursing world of to-day will weigh this subject, each one for themselves. Personally, I feel that the chronic sick need indeed a skilled nurse through years sometimes of pain and loneliness. It is in these cases that the true ideal of a nurse is put to the test. One fears that the surgical and exciting work sometimes tends to deaden the love and sympathy of the real woman, without which it is next to impossible to nurse the chronic patient. Remembering my own mother, surrounded with everything tending to her comfort save trained and skilled hands, and knowing how much she lacked in consequence, with all the earnestness I possess I would say to my fellow workers, "Never think you are wasting time when called to tend those appointed to die."

### DR. BURNET AND THE NURSING OF SICK CHILDREN.

"MATRON" writes:—In THE HOSPITAL of May 27 there appears an article on "The Nursing of Sick Children," by Dr. Burnet, in which he specially refers to the symptoms that a nurse should note and to the treatment a nurse should adopt in cases, first, of convulsions, and, secondly, of meningitis. After 12 years' experience as a nurse, may I criticise some of his remarks? Regarding the value of baths in the treatment of fits, he says it is the custom to place every child or infant who has a fit into a bath of "very hot water." No nurse of any experience would ever use "very hot water," but would employ a bath at temperature 100° Fahr., raising to a maximum temperature of 105° Fahr. after the child is in. Such a bath does not "serve to increase congestion" if at the same time cold wet clothes are applied to the head—an important item to which Dr. Burnet omits to refer. A hot bath properly given does not "excite the little patient or tend to bring on a succession of convulsive seizures." The other alternative which Dr. Burnet suggests, namely, "to place the feet in a warm bath to which a little mustard has been added, accompanied by a cold application to the head," is unsuitable. The term used, a "little mustard," is very vague, an ounce to every five gallons being the usual proportion, and should be mixed to a paste and added to the bath. To give such a bath the child must be in an upright posture, whereas a recumbent position seems more suitable for a child in a convulsion. In dealing with "meningitis," while referring to temperature, pulse, and respiration, Dr. Burnet omits to instruct the nurse to notice the condition of the "pupils," which is a very necessary point. He advises a soap and water enema "every other morning," but if bowel feeding has to be "resorted to" a daily morning enema of soap and water surely is necessary to obviate bowel irritation and the ejection of the nourishment. He suggests that the nurse should endeavour to keep the patient as dry as possible by using sheets of cotton-wool—a very dirty and expensive method. Constant attention to the "draw sheet" and mackintosh is, I think, the best way of keeping the patient dry. In dealing with "bed sores," Dr. Burnet omits to advise the thorough washing of the patient with soap and water, and the careful drying of the parts, before the application of methylated spirit. In "meningitis" the eyes of the patient are usually wide open, they therefore become dry, and are liable to be irritated by dust. Dr. Burnet makes no reference to the necessity of frequent laving of the eyes.

[There can be no doubt that hot baths do serve to increase cerebral congestion and are therefore to be avoided, apart altogether from the unnecessary disturbance which they occasion. A hot mustard foot bath can be given in the recumbent position perfectly well. The condition of the pupils in meningitis is, from a nursing point of view, quite unimportant. For relieving the constipation a soap and water enema every second day, as advised, is sufficient. If, however, rectal feeding is resorted to a simple hot water enema may be used every day. Dr. Burnet's remarks, however, had reference to the treatment of constipation. It is exceptional to find the eyes wide open in meningitis, save when the patient is moribund, and even then they may be found closed.—ED. THE HOSPITAL.]

### Appointments.

[No charge is made for announcements under this head, and we are always glad to receive and publish appointments. The information, to insure accuracy, should be sent from the nur es themselves, and we cannot undertake to correct official announcements which may happen to be inaccurate. It is essential that in all cases the school of training should be given.]

BRISTOL BLIND ASYLUM.—Miss Gummer has been appointed matron. She was trained at the Liverpool Royal Infirmary and has since been sister at Carnarvon and Anglesey Infirmary, matron of Stapleton Workhouse Infirmary, and night superintendent at Bristol General Hospital. She holds the certificate of the Central Midwives Board.

CITY HOSPITAL, LIVERPOOL, NORTH.—Miss Eileen M. Hendrick has been appointed sister. She was trained at the Wolverhampton and Staffordshire General Hospital, and was afterwards deputy sister of the Women's Medical and Gynæcological Wards. She has since been sister at the City Hospital, Lodge Moor, Sheffield.

GARFORTH ISOLATION HOSPITAL.—Miss E. G. Scaife has been appointed matron. She was trained at Crumpsall Infirmary, Manchester. She has since been at the York Home for Nurses; nurse at the South Eastern Fever Hospital, London; charge nurse at South Shields Union Infirmary; nurse in charge at Ripon Union Infirmary; charge nurse at the Hunslet Union Infirmary, Leeds; district nurse in Yorkshire; head nurse at the Wharfedale Union Infirmary; and district nurse at Moseley, near Manchester.

GLASGOW LOCK HOSPITAL.—Miss Margaret P. Love has been appointed charge nurse. She was trained at the Western Infirmary, Glasgow, and the Rotunda Hospital, Dublin. She has since been night superintendent at the Rotunda Hospital, Dublin, and has done holiday duty as night superintendent at the Salop Infirmary, Shrewsbury.

INVERNESS WORKHOUSE INFIRMARY.—Miss E. C. R. Philp has been appointed matron. She was trained at Edinburgh Royal Infirmary, and has since been assistant matron at the Royal Berkshire Hospital, Reading.

JOINT INFECTIOUS HOSPITAL, MITCHELL LAITHES, YORKSHIRE.—Miss Magdalen Cartwright has been appointed matron. She was trained at the Royal Alexandra Hospital, Rhyl, and the Royal Southern Hospital, Liverpool. She has since been head nurse at the Children's Hospital, Toxteth

Park, Liverpool, sister at the City Hospital, Birmingham, and head sister at the Infectious Hospital, Skipton.

Northumberland County Nursing Association.—Miss Anna R. Hunter has been appointed assistant superintendent. She was trained at Dundee Royal Infirmary and has since been Queen's nurse in Dundee and in Barry, South Wales; and nursing sister in the Concentration Camp Hospitals, South Africa. She received midwifery training at Gloucester and holds the certificate of the Central Midwives Board.

Northwood Hospital (Branch of Mount Vernon Hospital for Consumption, Hampstead).—Miss May Donaldson has been appointed matron. She was trained at the Great Northern Central Hospital, London, and has since been sister at the London Temperance Hospital.

Prudhoe Memorial Convalescent Home, Whitley Bay, Northumberland.—Miss Maude Mason has been appointed assistant-matron. She was trained at St. Bartholomew's Hospital, Rochester, and has since been charge nurse at the Brook Fever Hospital, sister at the Manchester Royal Eye Hospital, night superintendent at the General Hospital, Walsall, and lately she has been engaged in private nursing in Birmingham.

Royal National Hospital for Consumption for Ireland.—Miss G. M. Phillips has been appointed sister. She was trained at Radcliffe Infirmary, Oxford, and has since been sister at Chorlton Union Hospital, Manchester, sister in the Army Nursing Service, and sister at Shoreditch Infirmary.

Stockton-on-Tees District Nursing Association.—Miss Blower has been appointed lady superintendent and matron of the nurses' home. She has been for 15 years matron of the Ardwick District Nurses' Home, Manchester.

Strand Union Workhouse Sick Wards, Upper Edmonton.—Miss Alice Dedman has been appointed superintendent nurse. She was trained at Birmingham Poor-law Infirmary, and has since been nurse at St. John's Hospital, Leicester Square, London, and nurse at the Borough Sanatorium, Brighton.

Tredegar Park Cottage Hospital, Tredegar, Mon.—Miss Frances Holeister has been appointed matron. She was trained at Cardiff Infirmary, where she has since been staff nurse.

Willesden Workhouse Infirmary, Acton Lane.—Miss Jessie Charlotte Cole has been appointed charge nurse. She was trained at St. Pancras Infirmary, and has since been assistant nurse at Brook Fever Hospital, and charge nurse at Park Fever Hospital, London.

### Novelties for Nurses.

(By Our Shopping Correspondent.)

### BADGES.

Nurses' badges have become very popular of late. The similarity of uniforms, more especially of outdoor uniforms, renders a distinctive mark very suitable. At such gatherings as the Royal National Pension Fund Receptions and the Assemblies of the Royal British Nurses' Association, or of the Queen's Jubilee Nurses' Institute, the variety of the badges worn is both interesting and instructive. One of the latest badges introduced is that for Queen Alexandra's Imperial Military Nursing Service. The charming little badge (it is little more than an inch long) is made of silver and silver gilt. The crown and band which encircles the cross, and bears the title of the service, is in silver gilt, and so is the Queen's initial "A" in the centre of the cross. The cross is of silver with a white ground, and silver letters on a white ground are employed for the motto. The badge is made by Messrs. Gaunt and Son, of London and Warstone Parade, Birmingham, with whom this kind of work is a speciality.

They have sent other badges for inspection in plain silver, and silver and enamel, which are both strong and effective.

### A NURSE'S OUTFIT.

The Nurses' Outfitting Association, Stockport, have just issued an illustrated catalogue, which should prove of great interest to nurses, as it contains descriptions, and has prices plainly affixed, of a large and assorted number of nursing requisites. To mention a few of their lines which I have myself seen, I will commence with a nurse's dress made in double-warp zephyr, and of a dark butcher blue in shade. The body of the gown is lined throughout with material which before being made up is always shrunk; the zephyr itself appears exceedingly strong, and the whole dress is well and substantially made. The sleeves can be opened and turned up well above the wrist, and the price, which includes, if nurses wish, tucks in the bodice and skirt, is only 15s. I should say that a good feature of the dress consists in the hooks and eyes at the back for affixing the bodice to the skirt, and therefore ensuring a neat appearance. "Danco" serge, which can be had in dark blue, brown, green, or black, will be found good material for nursing cloaks, as it is fast dyed and shrunk, and does not change colour. It is 52 inches wide, and its price is 2s. 3d. per yard. A pretty little bonnet made in any coloured velvet, a fine pedal straw crown, and slightly pointed in front, is called the "Gladys," and is catalogued at 7s. 11½d., while the "Sister Helen," which might advantageously be a size larger, can be bought either in the same or in a cheaper quality. For those nurses who are partial to veils there is a many-hued assortment in all-silk "unspottable" gossamer. And last, I have to mention a tailor-made nurse's cloak, the "Sister Mary," in "Danco" serge. This is made with a single cape, and has a simple draw-string at the waist—in fact, its key-note is simplicity, and it is certainly delightfully cool and light for summer wear. The price is 25s.; and the Nurses' Outfitting Association will be pleased to forward their catalogue and patterns to any nurse who cares to write for them.

### AN ANTISEPTIC SOAP.

Those who have delicate skins should never use the coarse alkaline soaps which are commonly sold. All good makers recognise the necessity of providing soaps especially suited to those who have skin troubles. Messrs. Edward Cook have received a gold medal for their antiseptic soap which contains biniodide of three different strengths, according to the requirement of the purchaser. Preparations of iodine are not unpleasant, and the ½ per cent. biniodide soap is hardly distinguishable from a pleasantly-scented toilet soap. Messrs. Cook's toilet soaps are of first class quality and scented in good taste. The Dorina, Javon Mignora, and the "Throne" are especially to be recommended.

### A NEW PILLOW SUPPORT.

Mrs. Furnivall, of 13 Fawe Park Road, Putney, who is herself an invalid, draws attention to her contrivance to vary the position of invalids, more especially those who are long bed-ridden. Her arrangement is an upright post, with a horizontal arm at the top. This arrangement is attached to the back of the bed, and from it is suspended the pillow by stout cords, in such a manner as to allow of a frequent change in position of both head and shoulders of the patient.

### NURSERY MATTERS.

Trained nurses are so frequently in charge of the modern nursery, and they often take part in the selection of equipment and accessories; it is not therefore unsuitable to draw the attention of such members of the nursing profession to the illustrated catalogue of varied and elegant perambulators or baby cars issued by Messrs. Lloyd Courts and Co., Coventry.

### To Nurses.

We invite contributions from any of our readers, and shall be glad to pay for "Notes on News from the Nursing World," or for articles describing nursing experiences at home or abroad dealing with any nursing question from an original point of view according to length. The minimum payment is 5s. Contributions on topical subjects are specially welcome. Notices of appointments, letters, entertainments, presentations, and deaths are not paid for, but we are always glad to receive them. All rejected manuscripts are returned in due course, and all payments for manuscripts used are made as early as possible after the beginning of each quarter.

### TRAVEL NOTES AND QUERIES.

By our Travel Correspondent.

Excursion to St. Malo (A. M. W.).—Yes, take advantage of the excursion season. See answer to "Mab." You can do it at your higher figure. Hotels are quite beyond your purse, and are unusually expensive at St. Malo.

Oberammergau (A. A. G.).—Many thanks for kind information which is filed for future use. Unfortunately so few can afford so long a journey, but I am always glad to have information as to accommodation from a personal source.

By Rail all the Way (A. P.).—I do not quite know what you mean by "rail all the way." To make both ends meet you must go via Tower Bridge to Ostend by G.S.N.C., 9s. second class return. Then go to Bruges to Mrs. Dear, Pension Internationale, 4 Rue Anglaise. If you stay there all the time you can do it comfortably on the money. There is much to see in Bruges. Write to Mrs. D. on a prepaid return letter-card and ask her if she has a room for you at the time, and also ask her terms.

A Month at Grindelwald (Nurse A.).—The journey is not so expensive as you imagine. Second class return *via* Dieppe, Paris, and Berne is £5 12s. 3d. I do not think that your money will last for a month. Write on a prepaid foreign return letter-card to Pension Wolter, Pension Alpenruhe, and Pension du Lac. If they will take you for 6 francs (5s.) per day each, that would come to £7, roughly speaking. Add this to the cost of your journey and it only leaves you £2 7s. 6d. for tips, washing, excursions, and other expenses. I do not consider it nearly enough. Write to Dr. Lunn, 5 Endsleigh Gardens, W.C., for his tours, he has some that will come cheaper than this.

Fine Scenery in Switzerland (H.).—It is a matter of individual taste, and it is impossible to speak definitely on the subject. I may say, moreover, that Neuchâtel is not especially fine. You do not tell me what you can afford to pay (please always repeat statements in a second letter), so I can hardly advise. Pontresina is excessively dear, so is St. Moritz; at the latter place, however, there is one cheap hotel—the Central. How would you like the environs of Geneva and Lausanne? At the latter place the Pension Campart Route d'Ouchy might suit you. It is very moderate. I think the Lake of Geneva would be as great a change as you could have after the places you have already seen. Thanks for information—filed for reference.

Holiday in Belgium (A. M. F.).—You cannot do all you propose for £15, because you say it is necessary to go by the Dover route. The fare second-class return is £1 16s. 4d. I conclude you mean you each have £15. I should arrange thus:—Go to Antwerp, via Harwich; it is not a long passage; second-class single ticket, 15s. Go to Hôtel Grand Miroir, 56 Vieux Marché au Blé. Ask for rooms on third or four floor. Stay there three days, go on to Brussels and stay at Hôtel du Rhin, 14 Rue de Brabant. Stay four or five days according to the length of your bill—ask for it each day. From Brussels you can visit Mechlin and Louvain. Then go on to Bruges, for the rest of the time visiting Ghent on your way. At Bruges go to Hôtel Panier d'Or, or, if full, to Hôtel St. Amand, 5 Rue St. Amand. Return from Ostend to Dover; second-class single, 19s. 4d.

Three Weeks in Belgium (Matron).—You cannot go so far for the money you have. Dinant and Antwerp are very far from Brussels. Go by G.S.N.C. to Ostend—second-class return, 9s. From there go to Brussels—Hôtel du Rhin, 14 Rue de Brabant. Ask for rooms on third or fourth floor; stay a week. Take one day to visit Mechlin, another for Louvain. Then go to Ghent for three days—Hôtel Aux Armes de Zeelande. One day spend seeing Courtrai and Oudenarde and one at Alost. Then on to Bruges for the remainder of your time. Go to Mrs. Dear, Rue Cour de Grand. A tram from the station passes her house. Should all her rooms be occupied, go to Hôtel Panier d'Or in the market-place. Grant Allen's "Cities of Belgium" would give you the information you want and is only 3s. 6d. Read also Ouida's novel "Two Little Wooden Shoes," it gives so much local colour.

Three Weeks at Lucerne (Dublin).—Your best way is via Harwich, Antwerp, and Bale. Second-class return, £5 7s. 6d. If you can get accommodation at 7 frcs. per day three weeks would come to £6, that leaves you only, roughly speaking, £1 13s. for tips (a large item) and excursions. It is not enough. Either give yourself another £2 or stay only a fortnight. As there are four in the party you might make an arrangement for less than 7 frcs. Write to the following persons on prepaid foreign letter cards—English will do:—Hôtel Rütli, Pension Gesegnet Matt, and Pension Felsburg. All these are at Lucerne. Say there are four of you and ask if the proprietors will take you all for 150 francs per week. If this can be arranged it leaves you a margin. There are no such places as you imagine to be found in Lucerne at the tourist season. Failing this arrangement I should advise your writing to Dr. Lunn, 5 Endsleigh Gardens, W.C., and asking for particulars of his tours at the time needed.

A Holiday Alone (Mab).—Certainly you can go alone anywhere in Brittany, and you can stay a fortnight comfortably for the amount you can spend. Write to Messrs. Cook, Ludgate Circus, for information about their cheap trips to St. Malo starting every Saturday. You must have correct hours for tidal sailings. As you are alone you had better go to a pension, where you will be able to make friends. I advise you to write to Madame Pallot, Maison Mathias, St. Servan, France, on a prepaid reply foreign letter-card—you can write in English. Ask her if she can take you at the date you wish and her terms, which will be probably 7 frcs.—that is 5s. 10d. Use my name. St. Servan adjoins St. Malo. If Madame Pallot's house is full try Mrs. Dyott, Villa Olga, St. Malo, France. Her house is cheaper, but generally full. In neither place will you be lonely and you will find the Bretons most friendly and kind. On arriving, after passing the Customs (no difficulty), a steam tram passing the Custom House will put you down near to either house. There is an interpreter who meets all the English boats. Tell me how you have enjoyed your trip on your return. For excursions go to Dinan, Mont St. Michel, and Dinard. Unknown companions are a mistake

### Notes and Queries.

**REGULATIONS.**

**The Editor is always willing to answer in this column, without any fee, all reasonable questions, as soon as possible.**

**But the following rules must be carefully observed.**

**1. Every communication must be accompanied by the name and address of the writer.**

**2. The question must always bear upon nursing, directly or indirectly.**

**If an answer is required by letter a fee of half-a-crown must be enclosed with the note containing the inquiry.**

*Treatment.*

(97) I should be glad to know if there is any hospital or institute near Waterloo where they give the Dowsing radiant heat treatment and if the fees are reduced to nurses and what they are?—*Trained Nurse.*

Write to headquarters for all particulars. The address is The Dowsing Radiant Heat Company, 24 Budge Row, London, E.C.

*Holiday.*

(98) Can you give me the address of a home where a nurse could have a nice holiday at a small expense. The nurse is not able to afford much and has no friends that she can conveniently stop with. She would prefer not near the sea, but if possible near London.—*K. D.*

If you will write again, giving fuller particulars, we could give you the address of a nice private home in Rickmansworth.

*Medical Dictionary.*

(99) Will you kindly tell me of a good medical dictionary with treatment of cases for use?—*District Nurse.*

You would find the "Dictionary of Treatment," by Sir W. Whitla, Professor of Materia Medica, Queen's College, Belfast, very useful. The price is 16s., and it can be obtained from the Scientific Press, or any bookseller.

*Training.*

(100) I should be glad of advice with regard to the length of training necessary to qualify me for obtaining a Government post abroad or under the Colonial Nursing Institute. . . . Will you kindly let me have the address of the Colonial Nursing Institute?—*Tommy.*

A certificate of a three years' training in a recognised hospital is necessary. The address of the Colonial Nursing Association is the Imperial Institute, London, S.W. Write to the Honorary Secretary.

*Epilepsy.*

(101) I shall be very grateful if you will kindly inform me if you know of any home that receives cases of epilepsy either gratuitously or for a small charge. My son, aged 13 years, developed this complaint about 18 months ago. He is now under a local doctor and is exempted from further school attendance. Country air would do him so much good, but I know of no one to help me?—*J. W. E.*

Write to the Secretary of the National Society for the Employment of Epileptics, 12 Buckingham Street, Strand, W.C.

*Johannesburg.*

(102) Will you tell me whether it would be advisable to go out to South Africa nursing, and also give me the addresses of hospitals in Johannesburg where I might apply for a post. . . . I should be pleased to meet with a patient going out. I am a certificated nurse and would act in any capacity.—*Nurse A. L.*

You might write to the matron of the Johannesburg Hospital. Address P.O., Box 1050, Johannesburg. Also for particulars to the Lady Superintendent of the Nurses' Co-operative Society, The Gables, 72 Jeppe Street, Johannesburg, but the nursing outlook in South Africa is so far from bright just now that we can hold out small hope that they will employ you. With regard to the latter part of your letter, if you cannot hear of some one privately, the only way of finding employment for the voyage is by advertising, or by answering advertisements.

*Army Nurse.*

(103) I wish to become an army nurse. Kindly tell me to whom I should apply.—*F. P. S.*

Write for particulars to the Secretary, War Office, 68 Victoria Street, S.W.

**Handbooks for Nurses.**

| | Post Free. |
|---|---|
| "A Handbook for Nurses." (Dr. J. K. Watson.) ... | 5s. 4d. |
| "Nurses' Pronouncing Dictionary of Medical Terms." ... | 2s. 0d. |
| "Art of Massage." (Creighton Hale.) ... ... ... | 6s. 0d. |
| "Surgical Bandaging and Dressings." (Johnson Smith.) | 2s. 0d. |
| "Hints on Tropical Fevers." (Sister Pollard.) ... ... | 1s. 8d. |

Of all booksellers or of The Scientific Press, Limited, 28 & 29 Southampton Street, Strand, London, W.C.

### For Reading to the Sick.

"THOU VISITEST THE EARTH."

THE harp at Nature's advent strung
Has never ceased to play;
The song the stars of morning sung
Has never died away.

And prayer is made, and praise is given,
By all things near and far;
The ocean looketh up to heaven,
And mirrors every star.

The green earth sends her incense up
From many a mountain shrine;
From folded leaf and dewy cup
She pours her sacred wine.

The mists above the morning rills
Rise white as wings of prayer;
The altar-curtains of the hills
Are sunset's purple air.

The winds with hymns of praise are loud,
Or low with sobs of pain,—
The thunder-organ of the cloud,
The dropping tears of rain.

With drooping head and branches crossed
The twilight forest grieves,
Or speaks with tongues of Pentecost
From all its sunlit leaves.

*Whittier.*

"He shall come down like rain
Upon the mown grass."
Even so He came, and shall still come.

Three days ago the field, in its pageant of fresh beauty, with shimmering blades and tossing banners, greeted sun and shower alike with joy for the furtherance of its life and purpose; now, laid low, it hears the young grass whisper the splendour of its coming green; and the poor swathes are glad at the telling, but full of grief for their own apparent failure. Then in great pity comes the rain, the rain of summer, gentle, refreshing, penetrating, and the swathes are comforted, for they know that standing to greet or prostrate to suffer, the consolations of the former and the latter rain are still their own, with tender touch and cool caress. Then once more parched by the sun, they are borne away to the new service their apparent failure has fitted them for; and perhaps as they wait in the dark for the unknown that is still to come they hear sometimes the call of the distant rain, and at the sound the dry sap stirs afresh—they are not forgotten and can wait.

As I write the monastery bell hard by rings out across the lark's song. They still have time for visions behind those guarding walls, but for most of us it is not so. We let slip the ideal for what we call the real, and the golden dreams vanish while we clutch at phantoms: we speed along life's pathway, counting to the full the 60 minutes of every hour, yet the race is not to the swift nor the battle to the strong.

It is our prerogative to be dreamers, but there will always be men ready to offer us death for our dreams. And if it must be so let us choose death; it is gain, not loss, and the gloomy portal when we reach it is but a white gate, the white gate maybe we have known all our lives barred by the tendrils of the woodbine.

*M. Fairless.*

## Figures and Tables

**Figure f1:**
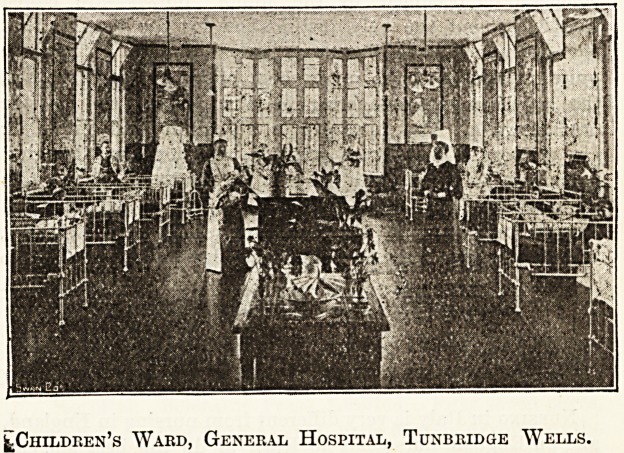


**Figure f2:**